# Synthetic Antimicrobial Peptides Fail to Induce Leucocyte Innate Immune Functions but Elicit Opposing Transcriptomic Profiles in European Sea Bass and Gilthead Seabream

**DOI:** 10.3390/md22020086

**Published:** 2024-02-14

**Authors:** Laura Cervera, Elena Chaves-Pozo, Alberto Cuesta

**Affiliations:** 1 Immunobiology for Aquaculture Group, Department of Cell Biology and Histology, Faculty of Biology, University of Murcia, 30100 Murcia, Spain; laura.cerveram@um.es (L.C.); alcuesta@um.es (A.C.); 2 Centro Oceanográfico de Murcia (COMU-IEO), CSIC, Carretera de la Azohía s/n, Puerto de Mazarrón, 30860 Murcia, Spain

**Keywords:** antimicrobial peptides (AMPs), NK-lysin, hepcidin, dicentracin, immunity, European sea bass, gilthead seabream

## Abstract

Antimicrobial peptides (AMPs) are promising molecules in diverse fields, including aquaculture. AMPs possess lytic effects on a wide range of pathogens, resulting in a potential replacement for traditional antimicrobials in aquaculture. In addition, they also have modulatory effects on host immune responses. Thus, the objective of this work was to evaluate the immunomodulatory capability of three known synthetic AMPs derived from European sea bass, NK-lysin (Nkl), hepcidin (Hamp), and dicentracin (Dic), in head-kidney cell suspensions from European sea bass and gilthead seabream. The tested peptides were neither cytotoxic for European sea bass nor gilthead seabream cells and failed to modulate the respiratory burst and phagocytosis activities. However, they modified the pattern of transcription of immune-related genes differently in both species. Peptides were able to promote the expression of marker genes for anti-inflammatory (*il10*), antiviral (*mx*, *irf3*), cell-mediated cytotoxicity (*nccrp1*, *gzmb*), and antibody responses (*ighm*) in European sea bass, with the Nkl peptide being the most effective. Contrary to this, the effects of those peptides on gilthead seabream mainly resulted in the suppression of immune responses. To conclude, European sea bass-derived peptides can be postulated as potential tools for immunostimulation in European sea bass fish farms, but more efforts are required for their universal use in other species.

## 1. Introduction

The discovery of antimicrobial peptides (AMPs) constitutes a promising field of research thanks to their potential applications in medicine, pharmaceutics, cosmetics, or animal production. Their complexity and variety of modes of action make AMPs excellent candidates as antimicrobial drugs claimed to solve the increasing problems with antimicrobial resistance [1], as they show low toxicity, biocompatibility, and a lack of resistance generation [2]. AMPs are structurally a very diverse group, but with some common features such as their short aminoacidic sequences (<50 aa), low molecular weight, positive charges, cationic and amphipathic residues, and a predominant structure in the alpha-helix [3]. The structure of AMPs determines their specificity and biological activity [2]. Thus, based on their structure, AMPs can be grouped into different families, such as defensins, hepcidins (Hamp), saponins, in which NK-lysin (Nkl) is included, or piscidins, which are only present in marine organisms and include dicentracin (Dic), among others [4]. Regarding their functional properties, a dual mode of action has been described: (1) AMPs possess direct lytic effects against a wide range of pathogens such as viruses, fungi, bacteria, or parasites [5]; and (2) AMPs can modulate host immune responses by promoting cell recruitment, modulating inflammatory responses, and bridging innate and adaptive responses [3]. 

Among the immunomodulatory actions triggered by AMPs, inflammation is the most characterized so far in fish. Generally, AMPs can regulate and control inflammatory responses without promoting exacerbated inflammation and avoiding tissue damage [6]. Nkl, Hamp, Dic, and other piscidins from different fish species are known to modulate the expression of cytokines with pro- or anti-inflammatory properties such as interleukin (IL)-1, IL-6, IL-8, tumor necrosis factor (TNF), IL-10, IL-22, or cyclooxygenase 2 (COX2), among others [7,8,9,10,11,12,13,14]. Besides, the role played by some AMPs, including Nkl, Hamp, or defensins, in the in vitro and in vivo induction of the interferon (IFN) type I pathway has been proposed in several fish species [7,15,16,17,18] and opens the door to their antiviral applications. In addition, several authors have documented that AMPs induce leukocyte trafficking and/or proliferation of B, T, and even NK-like cells by promoting cytokine or chemokine production, such as IL-21 or CXCR3, respectively [7,8,11,19]. Moreover, the capability of defensins or Hamp to bridge the innate to adaptive responses by inducing IgM production or promoting Toll-like receptors (TLR) or Myd88-dependent antigen recognition has been proposed in both mammals and fish [8,20,21,22,23]. In that sense, antigen recognition by the major histocompatibility complex might also be regulated by AMPs, as previous studies with piscidin and Nkl suggested in fish [9,12]. In fact, the role of AMPs in cell signaling has been extensively studied in humans [24]. Otherwise, the potential relationship between cell-mediated cytotoxicity (CMC) and AMPs has been proposed in mammals [25], but not in teleosts, even considering the evidence that related Nkl and fish CMC [26,27,28]. Finally, cross-regulation amongst AMPs has been described in European sea bass [11] and loach (*Misgurrus anguillicadatus*) [8,29]. Taking all this into account, AMPs are shown to play an important role in fish immunity responses though further and deeper characterization of their functions is required. 

In this context, we focused on three synthetic AMPs (Nkl, Hamp, and Dic) derived from European sea bass (*Dicentrarchus labrax*). Although the direct antibacterial activity of these peptides has already been demonstrated [30,31], their immunomodulatory activity has not yet been described. Thus, we aimed to investigate the in vitro effects on the innate immune responses of head-kidney (HK) cells from European sea bass, which remain unexplored. In addition, we investigated whether they could have the same biological functions in the HK cells of gilthead seabream (*Sparus aurata*) in order to explore a universal application. Their potential role as fish immunomodulators and applications in aquaculture will be discussed. 

## 2. Results

### 2.1. In Silico Predictions Show Homology between European Sea Bass and Gilthead Seabream Putative Peptides

**Figure 1:**
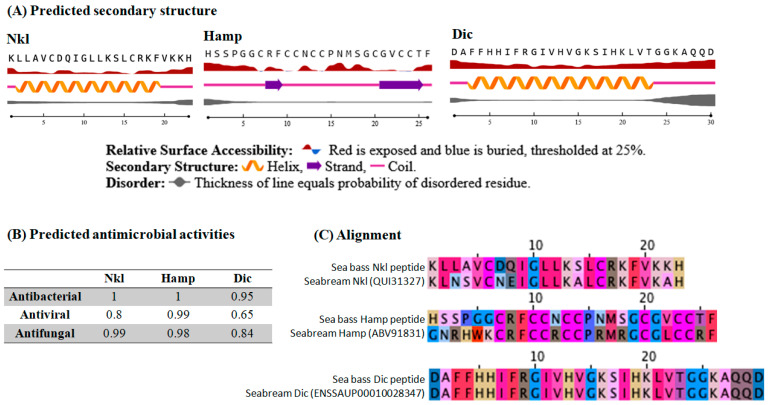
In silico analysis of the AMPs. (**A**) Secondary structures predicted for NK-lysin (Nkl), hepcidin (Hamp), and dicentracin (Dic) synthetic peptides by NetSurf P. (**B**) Antimicrobial activities predicted for Nkl, Hamp, and Dic synthetic peptides by AMPpred software. (**C**) Alignment between European sea bass synthetic peptides and gilthead seabream orthologues performed by Clustal Omega software and represented with Jalview.

Although direct lytic activity against bacteria or viruses has been demonstrated [30,31], we first performed an in silico analysis for comparisons. Regarding the predicted secondary structures, synthetic Nkl and Dic peptides presented a predominant alpha-helix structure, whilst Hamp showed coil-strand combinations (Figure 1A). Predictions for antimicrobial activity revealed that Hamp possesses the highest potential activity against bacteria, viruses, and fungi. For Nkl and Dic, the highest predicted values were always for antibacterial activity, while the weaker values were for antiviral activity, being lower for the Dic peptide (Figure 1B). The BLASTp analysis revealed high homology between the European sea bass-derived and synthetized peptides with the gilthead seabream putative orthologues, as demonstrated by the alignments (Figure 1C), suggesting similar functions. 

### 2.2. Nkl, Hamp, and Dic Synthetic Peptides Were Not Toxic for HK Cells

**Figure 2:**
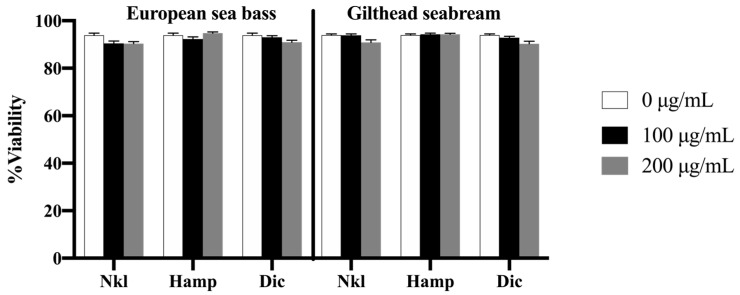
Viability of European sea bass or gilthead seabream head-kidney cells incubated with 0 (white bars), 100 (black bars), or 200 (grey bars) µg/mL of NK-lysin (Nkl), hepcidin (Hamp), or dicentracin (Dic) synthetic peptides for 6 h. Data represent the mean ± standard error of the mean (SEM) (*n* = 8).

We first evaluated if the AMPs were toxic for the European sea bass or gilthead seabream HK cells. The cell viability in the control (0 µg/mL group) was in all cases higher than 90%. The stimulation of HK cells of both species with the AMPs maintains an unaltered percentage of cell viability when compared to control cells (Figure 2).

### 2.3. Nkl, Hamp, and Dic Synthetic Peptides Failed to Modulate Innate Immune Activities

**Figure 3:**
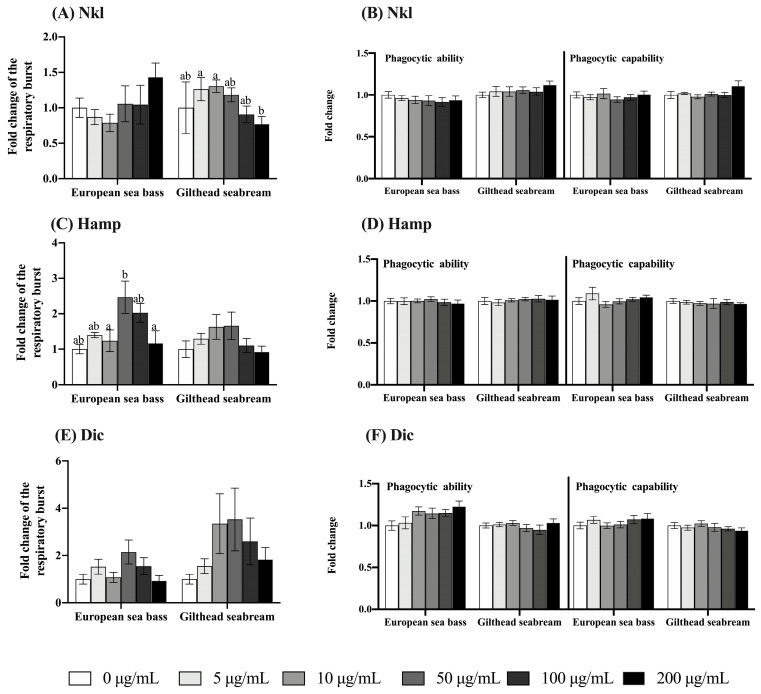
Respiratory burst (**A**,**C**,**E**) and phagocytic ability and capability (**B**,**D**,**F**) of European seabass or gilthead seabream head-kidney cells stimulated with 0, 5, 10, 50, 100, and 200 μg/mL of NK-lysin (Nkl), hepcidin (Hamp), and dicentracin (Dic) peptides for 6 h. Data represent the mean ± standard error of the mean (SEM) (*n* = 8). Different letters denote statistically significant differences among experimental concentrations by one-way ANOVA followed by a Tukey post hoc test (*p* < 0.05).

We next tested the immunomodulatory potential of Nkl, Hamp, or Dic peptides in the respiratory burst and phagocytic activities of HK cells. Our results show that neither the respiratory burst activity nor the phagocytic ability or capacity (Figure 3) of European sea bass and gilthead seabream HK cells were significantly altered by the AMPs when compared to the control cells (0 µg/mL group).

### 2.4. Nkl, Hamp, or Dic Synthetic Peptides Induced Species-Opposed Transcriptional Profiles

**Figure 4:**
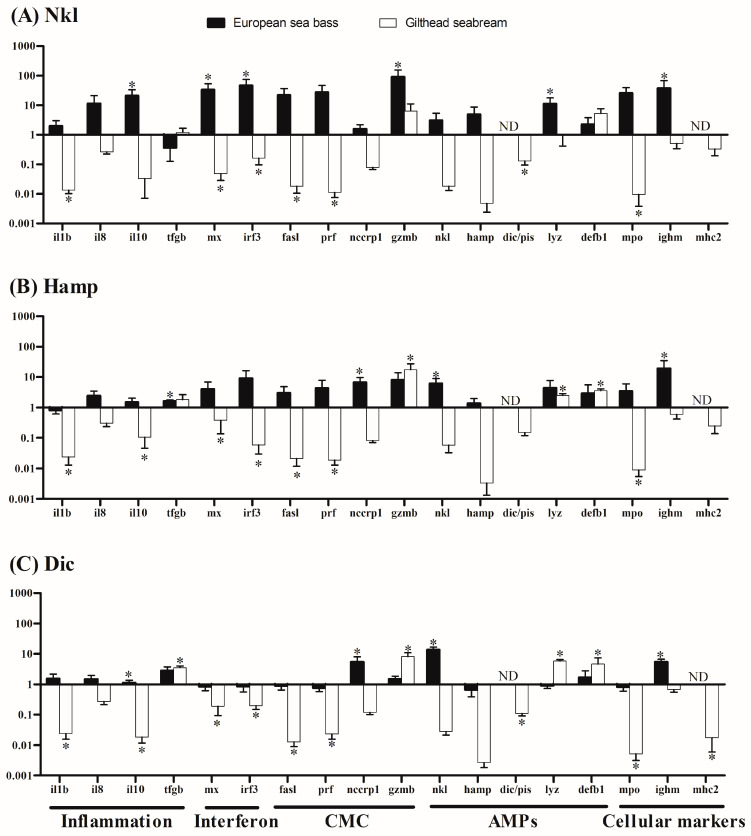
Gene expression of European sea bass or gilthead seabream head-kidney cells incubated with 50 μg/mL of NK-lysin (Nkl: **A**), hepcidin (Hamp: **B**), and dicentracin (Dic: **C**) synthetic peptides for 6 h. Data represent the mean ± standard error of the mean (SEM) (*n* = 6) of the fold change with respect to the control (0 μg/mL). Asterisks denote statistically significant differences between control and AMPs according to a Student’s *t*-test (*p* < 0.05). ND, non-detected.

Although we observed very little effect of AMPs on the innate immune activities analyzed, we evaluated the transcriptional profile of HK cells incubated with 50 µg/mL since this concentration provided the greatest differences with controls. In general, the synthetic AMPs produced opposed transcriptional patterns on sea bass and seabream HK cells (Figure 4). Thus, Nkl up-regulated the levels of European sea bass *il10*, *mx*, *irf3*, *gzmb*, *lyz*, and *ighm* transcripts, while the mRNA levels of *il1b*, *mx*, *irf3*, *fasl*, *prf*, *dic/pis*, and *mpo* were significantly reduced in gilthead seabream (Figure 4A). From its side, Hamp stimulated the transcription of *tgfb*, *nccrp1*, *nkl*, and *ighm* genes in European sea bass, whilst in gilthead seabream, those of *il1b*, *il10*, *mx*, *irf3*, *fasl*, *prf*, and *mpo* were down-regulated and *gzmb*, *lyz*, and *defb1* up-regulated (Figure 4B). Finally, in HK cells incubated with Dic peptide, the European sea bass expression of *il10*, *nccrp1*, *nkl*, and *ighm* genes was increased, while in gilthead seabream, the transcription of *il1b*, *il10*, *mx*, *irf3*, *fasl*, *prf*, *dic/pis mpo*, and *mhc2* genes was decreased and *tgfb*, *gzmb*, *lyz*, and *defb1 genes* increased (Figure 4C).

## 3. Discussion

Aquaculture is an extremely important economic sector but is facing serious difficulties, such as pathogen outbreaks leading to great losses for producers. Considering the scarce and limited availability of treatments without impact on the aquatic environment or public health, AMPs could constitute excellent tools for their use in aquaculture since their application in other fields has shown promising benefits [32,33]. Therefore, this paper has focused on the in vitro immunomodulatory potential of three synthetic AMPs since they seem to be good immunomodulators for fish [11,27,34,35] and increase the resistance against important marine pathogens both in vitro and in vivo [12,26,28,31,36,37,38]. The potential of the use of endogenous AMPs from a fish species to other fish species, or even humans, has been explored with excellent prospects [39,40,41,42,43]. In this sense, this manuscript also explores whether the employment of European sea bass-derived synthetic peptides resulted in the immunostimulation of another fish species, such as the gilthead seabream, apart from itself.

The first step in our study was to perform an in silico analysis since the sequence and secondary structures are of vital importance for their biological functions. The predominant alpha-helix structure in AMPs is tightly related to their bioactivity and antimicrobial functions and is stabilized by Alanine (A), Leucin (L), and Lysin (K) residues [44,45]. Synthetized Nkl and Dic peptides show an alpha-helix structure stabilized by multiple A, L, and K residues, conferring high antibacterial and antiviral activities [46], as the predictions pointed to. Otherwise, strand conformations, as predicted for Hamp, are known to contain two to ten cysteines, which form disulfide bridges [45]. This is characteristic of all the Hamp peptides and seems to be responsible for their activity [47,48]. Nevertheless, teleost Hamp peptides appear to be functional with or without these disulfide bonds, as described for the gilthead seabream synthetic peptide without these bridges [49] and European sea bass Hamp2 oxidated or non-oxidated peptides [30,43,50]. Finally, we compared the synthetized AMPs from European sea bass with the gilthead seabream orthologues, showing a high level of homology and conservation. Therefore, their biological functions might be similar. 

We checked and demonstrated that the incubation of European sea bass and gilthead seabream HK cells with synthetic Nkl, Hamp, and Dic peptides failed to be cytotoxic. The low toxicity of AMPs has been previously documented, pointing to this feature as a key factor for its use in several fields and among different species [51], and it also occurs in our study. Afterwards, we evaluated their immunomodulatory potential by exposing HK cells to a range of peptide concentrations from 5 to 200 μg/mL for 6 h, according to previous studies in other species [14,35,52,53,54]. Unfortunately, respiratory burst and phagocytic activities were unaltered by the tested AMPs. Similarly, mudskipper (*Boleophthamus pectinirostris*) macrophages exposed to 1 μg/mL Nkl for 8 h had unaltered phagocytic ability but increased bactericidal ability [13]. Otherwise, the ability of Nkl to enhance the respiratory burst has been described both at shorter (1 h of incubation with 1 μg/mL of peptide) and longer (12 h with 10, 20, 50, and 80 μM) stimulation times in barbel steed (*Hemibarbus labeo*) and in black rockfish (*Sebastes schlegelii*), respectively [14,53,54]. The notable differences observed among species might suggest that even if Nkl peptides are highly conserved, the immunomodulation elicited by each peptide can be species-dependent. In addition, Hamp stimulation of barbel steed leucocytes at a concentration of 10 μg/mL for 12 h resulted in the enhancement of the respiratory burst [52], while European sea bass and gilthead seabream HK cells incubated for 6 h did not. Further studies are needed to ascertain the functional immune responses elicited by AMPs in fish leucocytes.

Controlling the inflammatory response is a key aspect of the design of antimicrobial treatments to avoid exacerbated tissue damage [55,56]. Probably that is why most studies performed in teleost point to AMPs as important regulators of the inflammatory response at the gene expression level. In this framework, we next evaluated the transcription profile of several immune-relevant genes grouped into five functional categories (inflammatory molecules, type-I IFN response, cell-mediated cytotoxicity, AMPs, and leucocyte-type markers) using 50 μg/mL of the AMPs, as this dose showed the greatest differences with control cells according to our previous data. As regards Nkl, most authors have pointed to its pro-inflammatory actions [11,12,13,27,34]. However, our results suggest anti-inflammatory properties of Nkl in both fish species due to the significant up-regulation of *il10*, a cytokine that shows potent anti-inflammatory functions [57], and no other statistical differences observed in the expression of other cytokine genes, as also described in European sea bass upon an in vivo administration of Nkl peptides [15]. However, no consensus has been established as to whether pro- or anti-inflammatory responses are triggered by Hamp administration, since some authors have described the stimulation of pro-inflammatory cytokines [35,58,59], while others pointed to anti-inflammatory effects [11,19]. In this study, Hamp treatment up-regulated the anti-inflammatory markers in European sea bass and down-regulated the pro- and anti-inflammatory cytokines in gilthead seabream. Dic, however, had similar effects to Hamp in both species, but it also up-regulated the transcription of the anti-inflammatory cytokine, *tfgb*, in gilthead seabream. The anti-inflammatory properties of Dic and other piscidins have previously been described for both studied fish species [9,11]. So, in general, what we observed was the up-regulation of anti-inflammatory cytokines in AMP-treated HK cells of European sea bass, whilst in gilthead seabream the main effects were the down-regulation of pro-inflammatory cytokines. These data allow us to hypothesize that European sea bass-derived synthetic Nkl, Hamp, and Dic peptides might control exacerbated inflammatory responses in various fish species but employ different regulatory mechanisms depending on the species. Interestingly, the scarce modulation observed in the respiratory burst activity might be caused by the inhibition of the inflammatory response since both immune processes are tightly related [60]. 

Nkl, Hamp, and Dic have also been described as antiviral effectors in diverse species [27,31,36,37,61,62,63,64]. Thus, we evaluated the type-I IFN pathway since it is considered the main direct antiviral response in teleost. However, in European sea bass, only the Nkl peptide was able to induce the activation of IFN genes, as also occurred after in vivo administration in tongue sole (*Solea senegalensis*) and mudskipper [13,27], and after in vitro stimulation with beta-defensin in orange-spotted grouper (*Epinephelus coioides*) cells [65]. Nevertheless, as occurred in our study with gilthead seabream cells, Nkl peptides failed to induce the IFN pathway in other fish species [15,34], even when they showed protective capacity upon viral infection [12,66,67]. In contrast, our study showed that the IFN pathway was likewise unaltered by the administration of Hamp and Dic peptides to European sea bass HK cells, as also occurred when administering Hamp and Dic encoding plasmids in European sea bass [11], Hamp plasmids in loach, or Hamp peptide in medaka (*Oryzas latipes*) [29,63]. Similar to what happened with Nkl, Hamp and Dic greatly down-regulated the IFN markers in gilthead seabream cells, revealing a completely different pattern of expression in both species. Altogether, our data suggest that the antiviral properties of AMPs are not driven by the type-I IFN pathway, and other modes of action might be induced. 

CMC is involved in the elimination of virus-infected cells and related to the antiviral actions. So, we next evaluated several markers of the CMC in both species. To our knowledge, this study is the first to relate the modulation of CMC function or markers with AMPs. We have already documented the potential implication of *prf*, *fasl*, *gzmb*, or *nccrp1* in the European sea bass and/or gilthead seabream CMC and with the viral infections [28,68,69,70,71]. Likewise, induction of AMP-related genes upon viral infection in these species has also been described in our laboratory [37,62], suggesting an important connection between these two immune routes to fight against viruses, as suggested for humans [18,72]. Interestingly, all the studied synthetic peptides were able to modulate the transcription of one or several CMC marker genes in both species studied, reinforcing our initial hypothesis of the important connection between CMC and AMPs in the fish immune response. Thus, in European sea bass HK cells, all AMPs induce the transcription of CMC markers, as has also occurred in previous in vitro and in vivo studies in several species upon different pathogenic challenges [38,70,73,74] pointing to a potential synergic effect of AMPs and CMC in the infection resolution. By contrast, the transcription of *fasl* and *prf* was down-regulated in gilthead seabream HK cells by all AMPs studied, whilst Hamp and Dic peptides also increased the transcription of *gzmb*. These data point to a diminished CMC response in seabream leucocytes, which might be less efficient in cases of infection. 

The interplay among AMPs was also considered in this study. Previous data obtained in our lab described AMP cross-regulation in European sea bass [11], suggesting feedback among the expression levels of AMPs. In this work, we observed an up-regulation of *lyz* gene expression when European sea bass cells were treated with Nkl. Besides, Hamp administration to zebrafish (*Danio rerio*) resulted in the up-regulation of *lyz* gene expression [19]. Our data showed that in gilthead seabream cells, the transcription levels of *lyz* and *defb1* are up-regulated or those of *dic* down-regulated when treated with Hamp and Dic or Nkl and Dic, respectively. Taking all these into account, the idea of feedback among AMP expression is reinforced in this study. Although AMPs are mainly involved in innate immune responses, they can also serve as a bridge to adaptive immune responses [21]. To further evaluate this issue, we have studied the expression of a B-cell marker, *ighm*, obtaining a great up-regulation in European sea bass with all the studied AMPs and suggesting that they are able to stimulate adaptative responses. In sharp contrast to this, gilthead seabream HK cells stimulated with all tested AMPs kept this gene expression unaltered, but Hamp and Dic triggered the down-regulation of *mhc2*, which is also related to the adaptive response [9], suggesting a potential suppression of the adaptive response in gilthead seabream.

## 4. Materials and Methods

### 4.1. Animals

Healthy specimens of European sea bass (*Dicentrarchus labrax* L.; 131.4 ± 17.1 g body weight (bw); 20.9 ± 0.9 cm length) and gilthead seabream (*Sparus aurata* L.; 195.5 ± 29.1 g bw; 20.1 ± 0.8 cm length) were bred at *Centro Oceanográfico de Murcia*, *Instituto Español de Oceanografía* (COMU-IEO), CSIC, facilities and maintained in an open flow-through circuit with suitable aeration under natural water temperature and photoperiod. Fish were fed ad libitum with a commercial pellet diet (Skretting) with a maximum intake of 1% of their biomass. The environmental parameters, mortality, and food intake were recorded daily. Specimens were handled under the Guidelines of the European Union Council (2010/63/UE), the Bioethical Committees of the IEO (REGA code ES300261040017), and the approval of the Ministry of Water, Agriculture and Environment of the Autonomous Community Region of Murcia (Permit Number A13210701).

### 4.2. Peptide Design and Production 

European sea bass NK-lysin (Nkl; A0A218MG56), hepcidin 2 variant 1 (Hamp 2.1; KJ890397.1), and dicentracin (Dic; P59906) putative protein sequences were retrieved from databases. Sequences of the AMPs were designed, and the synthetic peptides were already probed for their antibacterial and/or antiviral direct activity [30,31]. AMPs were chemically synthesized by GeneScript (purity ≥ 90%) and shown in Table 1. AMPs were purchased as lyophilizates, so AMPs were first resuspended in culture medium at 1 mg/mL.

An in silico analysis was also performed. The prediction of the antimicrobial activity was carried out by the bioinformatic tool AMPpred (http://cabgrid.res.in:8080/amppred/result.php, accessed on 10 January 2024). Secondary structures were predicted at https://services.healthtech.dtu.dk/services/NetSurfP-2.0/ (accessed on 10 January 2024). The similarity between the European sea bass peptides and their gilthead seabream orthologues was also evaluated by Clustal Omega https://www.ebi.ac.uk/jdispatcher/msa/clustalo (accessed on 10 January 2024) and represented with Jalview viewer (version 2.11.3.0). 

### 4.3. Sampling and Head-Kidney Cell Isolation 

Fish (*n* = 14 European sea bass and *n* = 14 gilthead seabream) were anesthetized with clove oil (40 μL/L), weighed, exsanguinated from the caudal vein, and decapitated. Head-kidney cell suspensions, containing mostly leucocytes, were obtained by forcing the tissue fragment through a nylon mesh as previously described [75]. Cell suspensions were adjusted to 2 × 10^7^ cells/mL.

### 4.4. HK Cell Stimulation with AMPs 

Fifty μL of HK cell suspensions (1 × 10^6^ HK cells) were transferred into flat-bottomed 96-well plates, and the same volume of AMP dilutions was added to obtain final concentrations of 0 (control), 5, 10, 50, 100, or 200 μg/mL. Cells were incubated for 6 h at 25 °C. For viability, phagocytosis, and respiratory burst assays, we used 8 specimens (*n* = 8) for each species. For gene expression analysis, the cells from 6 specimens (*n* = 6/species) were stimulated with AMPs at a concentration of 50 μg/mL. In all cases, samples were processed individually and in triplicate.

### 4.5. Viability of Head-Kidney Cells

The viability of European sea bass and gilthead seabream HK cells was studied after incubation with 100 and 200 μg/mL of AMPs, as previously described [76]. For this, samples were mixed with 50 μL of propidium iodide (PI; 400 mg/mL; Sigma-Aldrich, St. Louis, MI, USA) before analysis in an FACScan flow cytometer (FACSCalibur™; Becton Dickinson, Franklin Lakes, NJ, USA) with an argon-ion laser adjusted to 488 nm. Analyses were performed on 5000 cells, which were acquired at a rate of 300 cells/s. Data were collected in the form of two-parameter side scatter (granularity, SSC) and forward scatter (size, FSC) dot plots and red fluorescence (FL2) histograms on a computerized system. Dead cells were estimated as the percentage of cells with propidium iodide (red-PI fluorescent cells). 

### 4.6. Respiratory Burst Activity

The respiratory burst activity of European sea bass and gilthead seabream HK cells after AMP stimulation was studied by a chemiluminescence method [77]. Briefly, samples were incubated with 100 μL of Hank’s balanced salt solution (HBSS) containing 1 mg/mL phorbol myristate acetate (PMA, Sigma-Aldrich, St. Louis, MI, USA) and 10^−4^ M luminol (Sigma-Aldrich, St. Louis, MI, USA). The plate was shaken, and luminescence was immediately read in a plate reader (BMG) for 1 h at 2 min intervals. The kinetics of the reactions were analyzed, and the maximum slope of each curve was calculated. Luminescence backgrounds were calculated using reagent solutions containing luminol but not PMA.

### 4.7. Phagocytosis

The phagocytic ability and capacity of European sea bass and gilthead seabream HK cells were studied by flow cytometry [78] using heat-killed and fluorescein isothiocyanate (FITC)-labeled yeast cells. Phagocytosis samples consisted of 60 μL of FITC-labeled yeast cells and 100 μL of AMP-treated HK cells. Samples were mixed, centrifuged (400× *g*, 5 min, and 22 °C), resuspended, and incubated for 30 min at 22 °C. At the end of the incubation time, samples were placed on ice to stop phagocytosis, and 400 μL of ice-cold PBS was added to each sample. The fluorescence of the extracellular yeasts was quenched by adding 50 μL of ice-cold trypan blue (0.5% in PBS). Standard samples of FITC-labeled yeasts or HK cells alone were included in each phagocytosis assay. All the samples were acquired in an FACScan flow cytometer set to analyze the phagocytic cells, which show the highest SSC and FSC values. Data on 5000 phagocytic cells were collected, and the phagocytic ability, defined as the percentage of phagocytic cells with one or more ingested yeast (green-FITC fluorescent cells), and phagocytic capacity, defined by the mean fluorescence intensity, equivalent to the relative number of ingested yeast cells per cell, were assessed.

### 4.8. Gene Expression Analysis

For the transcriptional study, an AMP concentration of 50 µg/mL was chosen. Total RNA from cell samples was isolated using the Quick-RNA mini prep kit (Zymo Research, Freiburg, Germany) and treated with DNAse I (0.9 U/mL; Zymo Research). The Tetro Reverse Transcriptase (Bioline) was used to synthesize the first-strand cDNA with oligo-dT from 1 µg of total RNA, at 25 °C for 10 min, 45 °C for 30 min, and 85 °C for 5 min. Real-time PCR (qPCR) was performed with a CFX96 Real-Time System (Biorad) using SYBR Green PCR Core Reagents (Applied Biosystems). Reaction mixtures were incubated at 95 °C for 2 min, followed by 40 cycles of 15 s at 95 °C, 1 min at 60 °C, and finally 15 s at 95 °C, 1 min at 60 °C, and 15 s at 95 °C. For each mRNA, gene expression was corrected by the elongation factor 1 alpha (*ef1a*) expression in each sample as 2^−ΔCt^, where ΔCt is determined by subtracting the gene expression of the *ef1a* Ct value from the target Ct [79]. The specific primers for the analyzed genes are shown in Table 2 and Table 3 and grouped into five categories: (1) inflammatory molecules: *il1b*, *il8*, *il10*, and *tfgb*; (2) type-I IFN response: *mx* and *irf3*; (3) cell-mediated cytotoxicity: *fasl*, *prf*, *nccrp1*, and *gzmb*; (4) AMPs: *nkl*, *hamp*, *dic*/*pis*, *lyz*, and *defb1;* and (5) leucocyte-type markers: *mpo*, *ighm*, and *mhc2*. Negative controls with no template were always included in the reactions.

### 4.9. Statistical Analysis

Statistical analyses were conducted using Graphpad Prism 8.2.1 (Boston, MA, USA) and SPSS v24.0 (New York, NY, USA) software. Data for immune parameters and gene expression are presented as fold changes with respect to the control group. Statistical differences in functional activities were determined by a one-way analysis of variance (ANOVA), followed by the Tukey post hoc test, whilst differences in gene expression were analyzed according to a Student’s *t*-test. The minimum level of significance was fixed at 0.05.

## 5. Conclusions

To conclude, our study showed the lack of toxicity and modulation of phagocytosis and respiratory burst innate activities of all studied synthetic AMPs in European sea bass and gilthead seabream head-kidney cells. However, AMPs showed strong immunomodulation at transcriptional levels, producing opposing expression profiles in the two fish species. In general, AMPs might increase in vitro the anti-inflammatory response, the antiviral functions of IFN, CMC, and antibody production in European sea bass leucocytes, making the Nkl peptide the most effective and promising for aquaculture applications. However, in gilthead seabream, the effects are contrary, promoting immunosuppression of the most studied immune responses. Further studies are mandatory to ascertain the potential immunomodulatory applications of AMPs in fish aquaculture and whether they could be universally used for different fish species. 

## Figures and Tables

**Table 1:** Peptide sequences used in this study.

Protein Name	Accession Number	Sequence
NK-lysin	A0A218MG56	KLLAVCDQIGLLKSLCRKFVKKH
Hepcidin 2.1	KJ890397.1	HSSPGGCRFCCNCCPNMSGCGVCCTF
Dicentracin	P59906	DAFFHHIFRGIVHVGKSIHKLVTGGKAQQD

**Table 2:** Primers for European sea bass used in this study for qPCR.

	Protein Name	Gene Name	Accession Number	Sequence (5’→3’)
Housekeeping	Elongation factor 1 alpha	*ef1a*	AJ866727	F: CGTTGGCTTCAACATCAAGA R: GAAGTTGTCTGCTCCCTTGG
Antiviral response	Interferon-induced GTP-binding protein Mx	*mx*	AM228977	F: GTATGAGGAGAAGGTGCGTCC R: CTCTTCCCCGAGCTTTGGTC
	Interferon regulatory factor	*irf3*	CBN81356	F: AGAGGTGAGTGGCAATGGTC R: GAGCAGTTTGAAGCCTTTGG
Leucocyte-type markers	Myeloperoxidase	*mpo*	DLAgn_0011834	F: GAAGAGTGGGGCCTTTGTTT R: CTGGGCCTCAGTGAAGACTC
	Immunoglobulin M heavy chain	*ighm*	FN908858	F: AGGACAGGACTGCTGCTGTT R: CACCTGCTGTCTGCTGTTGT
	Major histocompatibility complex 2	*mhc2*	AM113466	F: CAGAGACGGACAGGAAG R: CAAGATCAGACCCAGGA
Inflammation-related molecules	Interleukin-1 beta	*il1b*	AJ269472	F: CAGGACTCCGGTTTGAACAT R: GTCCATTCAAAAGGGGACAA
	Interleukin-8	*il8*	AM490063	F: GTCTGAGAAGCCTGGGAGTG R: GCAATGGGAGTTAGCAGGAA
	Interleukin-10	*il10*	DQ821114.1	F: ACTCCTCGGTCTCTTCTCCT R: TCCACAAAACGACAGCACTG
	Transforming growth factor beta	*tgfb*	XM_051399565.1	F: GCTACCATGCCAACTACTGC R: TGTTGCCTGCCCACATAGTA
Cytotoxic response	Fas ligand	*fasl*	ENSDLAT00005004342	F: GATGTGGGAGGAACCTGTGG R: GAACGGGTAGCTCTGGTCAC
	Perforin	*prf*	KY801204	F: CTGTACAACGGGCTTCTGGT R: ACTGGAGAACGTTGGACCAC
	Non-specific cytotoxic cell receptor protein 1	*nccrp1*	FM022070	F: TGGGGTGAGATACGTCCACT R: TGGTTTTGGTTGCTCTGACA
	Granzyme B	*gzmb*	DLAgn_00151210	F: AAGTTGAGCTCCAAGGCAAA R: TCCCCAGCCAGAGATGATAC
Antimicrobial peptides	NK-lysin	*nkl*	KY801205	F: GAAGAAACACCTCGGGGAAT R: GCAGGTCCAACATCTCCTTC
	Hepcidin	*hamp*	DQ131605	F: CCAGTCACTGAGGTGCAAGA R: GCTGTGACGCTTGTGTCTGT
	Dicentracin	*dic*	AY303949	F: GGCAAGTCCATCCACAAACT R: ATATTGCTCCGCTTGCTGAT
	Lysozyme	*lyz*	KJ433681.1	F: ATTTCCTGGCTGGAACACAG R: GAGCTCTGGCAACAACATCA
	Defensin beta 1	*defb1*	DLAgn_00041270	F: CCTTTCCTTGGTCTTGCCCA R: ACACACAGCACAAGAAGCCT

**Table 3:** Primers for gilthead seabream used in this study for qPCR.

	Protein Name	Gene Name	Accession Number	Sequence (5’→3’)
Housekeeping	Elongation factor 1 alpha	*ef1a*	AF184170	F: CTTCAACGCTCAGGTCATCAT R: GCACAGCGAAACGACAAGGGGA
Antiviral response	Interferon-induced GTP-binding protein Mx	*mx*	FJ490556, FJ490555	F: AAGAGGAGGACGAGGAGGAG R: CATCCCAGATCCTGGTCAGT
	Interferon regulatory factor	*irf3*	AM956899	F: TCAGAATGCCCCAAGAGATT R: AGAGTCTCCGCCTTCAGATG
Leucocyte-type markers	Myeloperoxidase	*mpo*	FM148574	F: TTGGTCCAGACATCCTCG R: ATGGGCAAAGCGGTAG
	Immunoglobulin M heavy chain	*ighm*	JQ811851	F: CAACATGCCCAATTGATGAG R: GGCACGACACTCTAGCTTCC
	Major histocompatibility complex 2	*mhc2*	DQ019401	F: CTGGACCAAGAACGGAAAGA R: CATCCCAGATCCTGGTCAGT
Inflammation-related molecules	Interleukin-1 beta	*il1b*	AJ277166	F: GGGCTGAACAACAGCACTCTC R: TTAACACTCTCCACCCTCCA
	Interleukin-8	*il8*	AM765841	F: GCCACTCTGAAGAGGACAGG R: TTTGGTTGTCTTTGGTCGAA
	Interleukin-10	*il10*	FG261948	F: AGGCAGGAGTTTGAAGCTGA R: ATGCTGAAGTTGGTGGAAGG
	Transforming growth factor beta	*tgfb*	AF424703	F: GCATGTGGCAGAGATGAAGA R: TTCAGCATGATACGGCAGAG
Cytotoxic response	Fas ligand	*fasl*	ENSSAUT00010022087	F: GCCACTTTGCCCGAACAATT R: GGTGGGGCAGTAAATCACCA
	Perforin	*prf*	XM_030407187	F: CTGGAGAAAGGCCTGTGGAG R: TCGGGCAACAGTCTTGGTTT
	Non-specific cytotoxic cell receptor protein 1	*nccrp1*	AY651258	F: ACTTCCTGCACCGACTCAAG R: TAGGAGCTGGTTTTGGTTGG
	Granzyme B	*gzmb*	AM957224	F: GAAACAAAGGAACGGGTCAA R: GAGCTGTCCATCTTTTGCTTG
Antimicrobial peptides	NK-lysin	*nkl*	MN240490	F: CGCACCTCGGAGAACTGATT R: TCCACGTCGCTTCGGTAAAA
	Hepcidin	*hamp*	CB184616	F: GCCATCGTGCTCACCTTTAT R: CTGTTGCCATACCCCATCTT
	Piscidin	*pis*	XM_030405214.1	F: GTGGCAACCGCAATAACACA R: AATGTTTGGCTGCAATGCGT
	Lysozyme	*lyz*	AM749959	F: CCAGGGCTGGAAATCAACTA R: CCAACATCAACACCTGCAAC
	Defensin beta 1	*defb1*	FM158209	F: CCCCAGTCTGAGTGGAGTGT R: AATGAGACACGCAGCACAAG

## Data Availability

Data will be made available on request.
